# Rapid Response Teams: addressing the evidence gap between high-income and low- and middle-income countries

**DOI:** 10.62675/2965-2774.20250239

**Published:** 2025-10-07

**Authors:** Marcio Manozzo Boniatti, João Gabriel Rosa Ramos, Regis Goulart Rosa, Sheila Nainan Myatra

**Affiliations:** 1 Postgraduate Program in Cardiology Universidade Federal do Rio Grande do Sul Porto Alegre RS Brazil Postgraduate Program in Cardiology, Universidade Federal do Rio Grande do Sul - Porto Alegre (RS), Brazil.; 2 Department of Critical Care Hospital de Clínicas de Porto Alegre Universidade Federal do Rio Grande do Sul Porto Alegre RS Brazil Department of Critical Care, Hospital de Clínicas de Porto Alegre, Universidade Federal do Rio Grande do Sul - Porto Alegre (RS), Brazil.; 3 Clínica Florence Salvador BA Brazil Clínica Florence, Salvador (BA), Brazil.; 4 Faculdade de Medicina Universidade Federal da Bahia Salvador BA Brazil Faculdade de Medicina, Universidade Federal da Bahia - Salvador (BA), Brazil.; 5 Department of Internal Medicine Hospital Moinhos de Vento Porto Alegre RS Brazil Department of Internal Medicine, Hospital Moinhos de Vento - Porto Alegre (RS), Brazil.; 6 Postgraduate Program in Pneumological Sciences Universidade Federal do Rio Grande do Sul Porto Alegre RS Brazil Postgraduate Program in Pneumological Sciences, Universidade Federal do Rio Grande do Sul - Porto Alegre (RS), Brazil.; 7 Department of Anaesthesiology, Critical Care and Pain Tata Memorial Hospital Homi Bhabha National Institute Mumbai Maharashtra India Department of Anaesthesiology, Critical Care and Pain, Tata Memorial Hospital, Homi Bhabha National Institute - Mumbai, Maharashtra, India.

Critical illness occurs in a continuum, and critical care must be delivered before, during, and after intensive care unit (ICU) hospitalization. First introduced in the medical literature in the early 1990s,^(1)^ Rapid Response Teams (RRTs) are designed to assess and manage patients exhibiting signs of acute clinical deterioration, as a way of giving access to critical care to acute patients outside of ICUs, with the primary goal of reducing in-hospital cardiac arrests and mortality among at-risk patients in general wards. Over time, as the implementation of RRTs has evolved and performance has improved, multiple studies – particularly of the before-and-after design – have provided evidence suggesting that these teams can effectively achieve these outcomes in mature healthcare systems.^(2)^ Adopting RRTs has been promoted worldwide and supported by healthcare quality and safety committees, becoming a widely endorsed component of hospital care. However, much of the existing evidence originates from high-income countries (HICs). A recent systematic review by Zhang et al.,^(3)^ which assessed the impact of RRTs on patient outcomes, found that 87% of the included studies were conducted in high-income settings, highlighting the imbalance of research contributions between high- and low-resource settings.

In this issue of Critical Care Science, Bianchini et al.^(4)^ present a scoping review aimed at mapping the current literature on RRT implementation in low- and middle-income countries (LMICs). Their findings underscore the persistent knowledge gap in this area and the resource constraints faced by health systems in LMICs. The available studies are concentrated in a few countries, exhibit considerable methodological heterogeneity, and frequently lack comprehensive data. Although there has been a recent increase in publications on this topic, the volume and quality of research in LMICs remain substantially behind that of HICs.

This issue warrants careful attention. Given the substantial contextual differences, assuming that evidence generated in HICs can be directly applied to LMICs with equivalent effectiveness would be unrealistic. Variations in hospital infrastructure, ICU availability, patient populations, staff expertise, and care processes can significantly influence the effectiveness of interventions. As such, evaluating interventions within the specific settings where they are to be implemented is critical, rather than assuming that findings from HICs will translate seamlessly to LMICs. Due to limited resources, LMICs have traditionally prioritized acute care management over proactive strategies to prevent clinical deterioration, mainly out of concern that preventive efforts might further strain already overburdened systems. This presents a paradox, since timely interventions like RRTs may alleviate pressure on acute care by preventing emergencies requiring more intensive and costly resources. Raising awareness of the potential benefits of RRTs in LMICs is essential to shifting perceptions and promoting a broader paradigm change in managing health care emergencies in these settings. In the case of RRTs, for instance, LMICs typically have fewer ICU beds per 100,000 inhabitants than HICs, leading to more pronounced bed shortages and a greater need for advanced clinical support within general wards. This scenario may amplify the potential impact of RRTs.

Additionally, differences in team composition, cultural attitudes toward end-of-life care, and the variable adoption of technologies such as electronic alerts further widen the gap between the realities of HICs and LMICs. These contextual differences underscore the importance of generating local evidence to guide context-appropriate implementation of RRTs. Moreover, implementation in LMICs may be further challenged by structural barriers such as workforce constraints, limited access to training, and infrastructure deficits, which can significantly affect the effectiveness and sustainability of RRTs in these settings (Figure 1).

**Figure 1:**
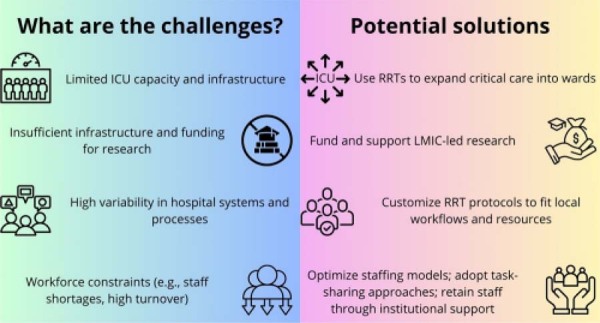
Challenges and potential solutions for implementing Rapid Response Teams in low and middle-income countries. ICU - intensive care unit; RRT - Rapid Response Team; LMIC - low and middle-income countries.

Enhancing research output from LMICs remains a critical challenge. Patients in these settings are consistently underrepresented in extensive international studies, and few investigations are led or conducted primarily within LMIC contexts.^(5)^ Addressing this imbalance requires targeted efforts: expanding funding opportunities, strengthening local research infrastructure, and building equitable partnerships with researchers from HICs – ideally under the leadership of LMIC institutions. Effective knowledge translation from HICs to LMICs is essential to improving outcomes for critically ill patients. This is particularly true in areas in which context matters deeply, such as the design and implementation of rapid response systems. There is an urgent need for locally driven studies that examine the optimal composition and training of RRTs, identify barriers and facilitators to timely activation, assess the role of electronic alert systems, and explore strategies for sustainable integration into existing health systems. Interventions developed in HICs cannot simply be transplanted into LMICs without adaptation. Recognizing the knowledge gap is an important first step but not enough. Bridging this divide will require coordinated action and long-term commitment from academic institutions, professional societies, and funding agencies. The path forward is challenging, but essential. Improving the care of critically ill patients in LMICs depends on generating new evidence and ensuring that this evidence is contextually relevant, accessible, and actionable. This review published in Critical Care Science by presenting the current evidence, acknowledging the limitations, proposing a core dataset to evaluate the implementation of RRTs, and suggesting new studies is an important contribution in this direction.

## References

[1] Daffurn K, Lee A, Hillman KM, Bishop GF, Bauman A (1994). Do nurses know when to summon emergency assistance?. Intensive Crit Care Nurs.

[2] De Jong A, Jung B, Daurat A, Chanques G, Mahul M, Monnin M (2016). Effect of rapid response systems on hospital mortality: a systematic review and meta-analysis. Intensive Care Med.

[3] Zhang Q, Lee K, Mansor Z, Ismail I, Guo Y, Xiao Q (2024). Effects of a Rapid Response Team on patient outcomes: a systematic review. Heart Lung.

[4] Bianchini L, Araújo LM, Jones D, Besen BA (2025). Rapid Response Teams in low and middle-income countries: a scoping review. Crit Care Sci.

[5] Salluh JI, Besen BA, González-Dambrauskas S, Ranjit S, Souza DC, Veiga VC (2024). Closing the critical care knowledge gap: the importance of publications from low-income and middle-income countries. Crit Care Sci.

